# Differential Proteomics and Functional Research following Gene Therapy in a Mouse Model of Leber Congenital Amaurosis

**DOI:** 10.1371/journal.pone.0044855

**Published:** 2012-08-31

**Authors:** Qinxiang Zheng, Yueping Ren, Radouil Tzekov, Yuanping Zhang, Bo Chen, Jiangping Hou, Chunhui Zhao, Jiali Zhu, Ying Zhang, Xufeng Dai, Shan Ma, Jia Li, Jijing Pang, Jia Qu, Wensheng Li

**Affiliations:** 1 Eye Hospital, Wenzhou Medical College, Wenzhou, China; 2 The Roskamp Institute, Sarasota, Florida, United States of America; 3 Department of Ophthalmology, The Second Affiliated Hospital of Kunming Medical College, Kunming, China; 4 Department of Ophthalmology and Visual Science, Yale University School of Medicine, New Haven, Connecticut, United States of America; 5 Department of Ophthalmology, University of Massachusetts Medical School, Worcester, Massachusetts, United States of America; 6 Department of Ophthalmology, College of Medicine, University of Florida, Gainesville, Florida, United States of America; 7 Neurobiology-Neurodegeneration and Repair Laboratory, Retinal Cell Biology and Degeneration Section, National Eye Institute, National Institutes of Health, Bethesda, Maryland, United States of America; University of Regensburg, Germany

## Abstract

Leber congenital amaurosis (LCA) is one of the most severe forms of inherited retinal degeneration and can be caused by mutations in at least 15 different genes. To clarify the proteomic differences in LCA eyes, a cohort of retinal degeneration 12 (*rd12*) mice, an LCA2 model caused by a mutation in the *RPE65* gene, were injected subretinally with an AAV vector (scAAV5-smCBA-hRPE65) in one eye, while the contralateral eye served as a control. Proteomics were compared between untreated rd12 and normal control retinas on P14 and P21, and among treated and untreated rd12 retinas and control retinas on P42. Gene therapy in *rd12* mice restored retinal function in treated eyes, which was demonstrated by electroretinography (ERG). Proteomic analysis successfully identified 39 proteins expressed differently among the 3 groups. The expression of 3 proteins involved in regulation of apoptosis and neuroptotection (alpha A crystallin, heat shock protein 70 and peroxiredoxin 6) were investigated further. Immunofluorescence, Western blot and real-time PCR confirmed the quantitative changes in their expression. Furthermore, cell culture studies suggested that peroxiredoxin 6 could act in an antioxidant role in *rd12* mice. Our findings support the feasibility of gene therapy in LCA2 patients and support a role for alpha A crystallin, heat shock protein 70 and peroxiredoxin 6 in the pathogenetic mechanisms involved in LCA2 disease process.

## Introduction

Leber congenital amaurosis (LCA) is an inherited retinal degenerative disorder characterized by severe loss of vision at early age. Affecting around 1 in 80,000 of the population, comprising 5% of the total inherited retinal degenerative diseases, LCA is the major cause leading to binocular blindness in children (10%∼20%) [1], [2]. Besides visual impairment from infancy, LCA is also typically characterized by nystagmus, sluggish or no pupillary responses and, low or even non-recordable electroretinogram (ERG) amplitude. LCA is regarded as an autosomal recessive disease, although some autosomal dominant cases were also reported [3], [4]. LCA has been linked to at least fifteen genes, which are *AIPL1*, *CRB1*, *CRX*, *GUCY2D*, *LRAT*, *TULP1*, *RPE65*, *RPGRIP1*,*CEP290*, *RDH12*, *LCA5*, *TULP1*, *RD3*, *IMPDH1* and *SPATA7* (RetNet:http://www.sph.uth.tmc.edu) [5]. About 16% of all LCA cases are caused by mutations of *RPE65* gene [6], which is mainly expressed in retinal pigment epithelium (RPE) [7]. *RPE65* is an isomerohydrolase in the canonical retinoid visual cycle, which is the enzymatic pathway that regenerates photoreceptor chromophore 11-*cis*-retinal after it is bleached in the process of light perception [8]. Studies in knockout mice (*RPE65 −/−*) demonstrated that the lack of *RPE65* protein does not allow a physiological recovery of 11-*cis*-retinal and rods and cones degenerate quickly after birth, probably because of the constitutive opsin signaling [9], while administration of 9-*cis* or 11-*cis*-retinal could partially restore both rod and cone function [10]. To date, the most successful example of experimental gene therapy for an ocular disease is the gene delivery of *RPE65* gene in LCA mice and patients [11]. rAAV-vector-mediated *RPE65* gene replacement has rescued morphological, biochemical and electrophysiological abnormalities present in murine models with *RPE65* deficiency [12], [13]. More importantly, several groups have reported rescue of vision after rAAV-vector-mediated gene replacement in the Swedish Briard dog, a spontaneous *RPE65*-null model [14]–[17], where stable vision improvement has been maintained over 8 years after a single rAAV vector administration [18], [19]. These results, in addition to the absence of side effects after rAAV vector subretinal delivery in non-human primates [20], have paved the way to ongoing clinical trials using rAAV2/2 vectors for *RPE65* gene replacement in patients affected by LCA due to *RPE65* mutations [21]–[24]. This form of LCA is particularly suitable for gene therapy because *RPE65* patients have a relatively preserved retinal morphology, despite severe and early vision impairment [25]. The clinical trial results in 15 children and adults followed up to 3 years are indeed promising and constitute the first successful example of gene therapy for inherited ocular diseases [26].

Meanwhile, very little is known about protein changes after gene therapy in retinal disorders. Lai et al. [27] and Bennicelli et al. [16] reported *RPE65* protein expression after the administration of a AAV2-RPE65 gene. Pawlyk et al. showed that subretinal injection of recombinant AAV vector packaged with a *RPGRIP* expression cassette into the *RPGRIP−/−* mouse eye, led to normal *RPGRIP* expression and localization [28]. Kostic et al. found that subretinal injection of a lentiviral vector driving expression of *RPE65* in *RPE65* deficient (R91W/R91W) mice, not only slowed down the cone degeneration, but also restored the cone-specific protein expression [29].

However, to the best of our knowledge, a study on the totality of protein expression before and after gene intervention has not been reported. Using the technique of two-dimensional electrophoresis (2-DE) and mass spectrometry, the present study analyzed retinal proteomic differences after subretinal injection of scAAV5-smCBA-hRPE65 in *rd12* mice, screened the proteins relevant to the retinal degeneration progression for further verification, and conducted cell culture experiments to test the important role of selected proteins, aiming to clarify the role of regulating factors in photoreceptor apoptosis and possible retinal protective factors for LCA and other related retinal degenerative disorders.

## Materials and Methods

### Gene therapy

Sixty mice of *rd12* (*Rpe65rd12*, or B6(A)-*Rpe65rd12*/J) mice were obtained from the Jackson Laboratory (Bar Harbor, ME), and 42 age-matched C57BL/6J mice were obtained from Animal Center of Wenzhou Medical College. All mice were bred and maintained in the Animal Facilities of Wenzhou Medical College (SPF). They were kept in a 12-hour light 12-hour dark cycle with an ambient light intensity of 18 lux and with free access to food and water. All experiments were approved by the Wenzhou Medical College’s Institutional Review Board and were conducted in accordance with the ARVO Statement for the Use of Animals in Ophthalmic and Vision Research. Three groups were assigned in the experiment: the treated right eyes of *rd12* mice were treated *rd12* group, the contralateral untreated left eyes were untreated *rd12* group and the age-matched wide-type C57BL/6J mice were the normal control group. The same scAAV5-smCBA-hRPE65 vector as we used in the previous studies in our lab was used in this experiment [30]. Subretinal injections were also performed with the previously described methods [12]. After pupil dilation and general anesthesia, animals were prepared for treatment. A small incision within the pupil area was made through the nasal cornea with a 30.5-gauge disposable needle. A 33-gauge, unbeveled, blunt needle mounted on a 5-μL syringe (Hamilton Co., Reno, NV) was introduced through the corneal opening, avoiding the lens and penetrating the neuroretina to reach the subretinal space in the inferior central region. One microliter of vector suspension (1×10^13^ genome containing particles/mL) containing 1% fluorescein was injected slowly into the subretinal space of the right eye. Injections were always performed in the right eye, leaving the uninjected left eye as a control. The injected retinal area was visualized by fluorescein positive subretinal blebs demarking the retinal detachment. After the injection procedure, 1% atropine eye drops and 0.3% tobramycin-dexamethasone eye ointment (Alcon Laboratories Inc., Fort Worth, TX) were given 3 times each day for 3 days. Only injected mice that had more than 95% retinal detachment after the subretinal injection and with minimal complications were kept for further evaluation. Animals with any of the following serious surgical complications, including iris-cornea adhesion, iris or retinal hemorrhage, and damage to the lens which could cause cataract formation, were excluded from the experiment. The contralateral untreated *rd12* eyes and the age-matched normal C57BL/6J eyes were used as controls.

### Electroretinograms

Scotopic and photopic ERGs of the 3 groups at age P14, P21 and P42 were recorded as published previously [30]. Briefly, full-field ERGs were recorded with a custom-built Ganzfeld dome connected to a computer based system (Q450SC UV; Roland Consult, Wiesbaden, Germany). LED stimuli of 6 intensities ranging from −35, −25, −15, −5, 5 to 15 cd⋅s/m^2^, were used under scotopic conditions. White LED stimuli of two intensities (1 cd⋅s/m^2^, 1.96 cd⋅s/m^2^) were used to generate and record cone responses under photopic conditions, with a background white light at 30 cd/m^2^. The signal was amplified 1000-fold and bandpass filtered between 1 and 100 Hz. After dark adaption overnight, scotopic ERG was recorded between 8AM to 11AM, which was followed by photopic ERG. Recordings were done at age P14, P21 and P42. All testing was performed in a climate-controlled, electrically isolated dark room under dim red light illumination. Systemic anesthesia was achieved by the intraperitoneal administration of a mixture of ketamine (72 mg/kg) and xylazine (4 mg/kg), while cornea was anesthetized by a drop of 0.5% proparacaine hydrochloride, and pupils dilated with 1% atropine and 2.5% phenylephrine hydrochloride. Small amount of 2.5% methylcellulose gel was applied to the eye, and a special Ag/AgCl wire loop electrode was placed over the cornea as an active electrode. Needle reference and ground electrodes were inserted into the cheek and tail, respectively. Recordings were started from the lowest light intensity to the highest. Body temperature was maintained by placing the animals on a 37°C warming pad during the experiment.

### Two-Dimensional Electrophoresis

2-DE was performed as previously described [31]. Briefly, retinas were mechanically dissected from enucleated eyes, homogenated and then mixed with 100 μl of lysis buffer (7 M urea, 2 M thiourea, 4 % CHAPS, 2 M TBP, 20 mM Tris, 1% IEF buffer, 1 mM PMSF, 100 μg/ml DNase, 100 μg/ml RNase), and centrifuged at 15,000 rpm for 1 minutes at 4°C. The protein concentrations for the samples were measured using the Bradford method. Protein samples were resuspended in IEF buffer (7 M urea, 2 M thiourea, 4% CHAPS, 65 mM DTT, 1% IEF buffer and 0.001% bromophenol blue). Equal amount of protein samples from the retina (100 μg) was applied to a 17 cm PH 3.0–10.0 2-DE gel. For the first dimension, IEF was performed using the following voltage program: 150 V for 1.5 h, 250 V for 1.5 h, 500 V for 1.5 h, 1000 V for 2 h, 5000 V for 3 h, 7000 V for 2 h, 10000 V for 5 h, 500 V for 20 h. The strip after IEF was equilibrated for 15 min in the equilibrium buffer I including DTT, then for another 15 min in the equilibrium buffer II including iodoracetamide. In the second dimension, 13% sodium dodecyl sulfate (SDS) gels were run at 12 mA per gel 30 min and then 24 mA per gel to the end. The electrophoresis was stopped when the bromophenol blue front had traversed the gel. The completed 2-D gels were stained with silver nitrate. Comparisons on P14, P21 were made between untreated *rd12* and normal control retinas, and those on P42 were among treated and untreated *rd12* retinas and control retinas. Differential protein spots were excised from the gels and destained in 200 mM ammonium bicarbonate/50% acetonitrile (ACN) (1∶1). Gel pieces were dried in vacuum and then were digested into peptides using trypsin overnight at 37°C.

### Mass Spectrometry Protein Identification

Preparation and analysis of protein spots for mass spectrometry were performed using a previously described method [31]. Briefly, matrix-assisted laser desorption ionization time-of-flight (MALDI-TOF) was performed to obtain peptide mass fingerprints on a QSTAR XL quadrupole-TOF mass spectrometer (Applied Biosystems Inc., Foster City, CA) Peptide peaks were submitted to Mascot software (Matrix Science Inc., Boston, MA) to obtain initial protein identification. Positive identification was based on a significant Molecular Weight Search (MOWSE) score and that the mass tolerance was less than 50 parts per million. Confirmation of initial identities was obtained by peptide mass sequencing using either MALDI or electrospray ionization (ESI) MS. Peptides analyzed by ESI were separated by liquid chromatography online using QSTAR Pulsar quadrupole TOF (Applied Biosystems Inc.) and ionized as described.

### Immunofluorescence

Frozen sections were prepared from freshly enucleated eyes. Then the sections were briefly washed with 0.01 M phosphate-buffered saline (PBS), the retinas were incubated with 5% normal goat serum (NGS) blocking solution for 1 hour, and then stained with rabbit anti-HSP70 (1∶100) or anti-PRDX6 (1∶200) or anti-CRYAA (1∶200) overnight at 4°C. Antibodies for PRDX6 were from Abcam (Cambridge, MA), and those for HSP70 and CRYAA were from Santa Cruz Biotechnology. After the exposure to primary antibodies, the sections were washed in 0.01 M PBS, DAPI(1∶50)was added for 5 minutes, and then the sections were re-washed with PBS. After sealing, sections were photographed with a fluorescence microscope (Axio Imager Z1; Carl Zeiss Meditec, Oberkochen, Germany). Images were taken predominantly from the inferior central retina.

### Western blot

Retinal tissue was washed with cold PBS and subjected to lysis in a lysis buffer (50 mM Tris-Cl, 1 mM EDTA, 20 g/L SDS, 5 mM dithiothreitol, 10 mM phenylmethylsulfonyl fluoride). Then a BCA™ Protein Assay Kit (Pierce Inc., Rockford, IL) was used to test the protein concentration. Equal amounts of protein (20 μg each) and rainbow molecular weight markers (Amersham Pharmacia Biotech, Amersham, UK) were separated by 12% SDS-PAGE, then electrotransferred to nitrocellulose membranes. Membranes were blocked with a buffer containing 5% nonfat milk in PBS with 0.05% Tween 20 for 2 hours and incubated overnight with antibody at 4°C. After a second wash with PBS containing 0.05% Tween 20, the membranes were incubated with peroxidase-conjugated secondary antibodies (Santa Cruz Biotechnology, Santa Cruz, CA) and developed with an enhanced chemiluminescence detection kit (Pierce, Inc.). Glyceraldehyde-3-phosphate dehydrogenase (GAPDH) was used as a loading control. Antibodies for CRYAA, HSP70 and PRDX6 were the same to those used in immunofluorescence.

### Real-time PCR

Total RNA was extracted from retina (Trizol reagent; Invitrogen-Gibco, Grand Island, NY) and 0.2 μg of RNA from each sample was reverse transcribed with M-MLV reverse transcriptase according to the manufacturer's instructions (Promega, Madison, WI). The primers were designed using Primer Express 3.0 software (Applied Biosystems, Inc., Foster City, CA), and the sequences and product length are provided in Table 1. PCRs were performed 7500 Real-Time PCR System (Applied Biosystems), with 2×SYBR® Green PCR Master Mix (Applied Biosystems). The results were normalized to the housekeeping gene GAPDH, with the mRNA levels standardized in every group and condition.

**Table 1:** Primers for HSP70, PRDX6, CRYAA and β-actin in real-time PCR.

Genes	sense primer 5′–3′	antisense primer 5′–3′	length bp
HSP70	TGGTGCTGACGAAGATGAAG	AGGTCGAAGATGAGCACGTT	253
PRDX6	CGCCAGAGTTTGCCAAGAG	TCCGTGGGTGTTTCACCATTG	115
CRYAA	ACAACGAGAGGCAGGATGAC	AGGGGACAACCAAGGTGAG	249
β-actin	CTACAATGAGCTGCGTGTGG	ACCAGAGGCATACAGGGACA	169

### Reactive Oxygen Species Test

Reactive oxygen species (ROS) test was conducted with fresh specimens. Immediately, the eye was embedded by OCT, then frozen for sections which were made 10 μm in thickness. Then the ROS test was performed using ROS Fluorescein Assay Kit (Genmed, USA), following the kit instructions. Briefly, the sections were washed with precooled cleaning liquid. Then they were treated with preheated stains and kept in humidified incubator at 4°C for 20 minutes. Finally, they were rewashed with cleaning liquid. After mounting, the sections were observed under fluorescence microscope.

### TUNEL Test for Apoptosis

Detection of degraded DNA fragments by Terminal Transferase nick-end-labeling (TUNEL) is a commonly-used method for quantitation of apoptosis. TUNEL Apoptosis Detection Kit (Roche, Switzerland) was used to detect retinal cell apoptosis following the kit instructions. Cryostat sections were incubated in 2% TDT (Roche) in TDT buffer (Roche) and 0.03% fluorescein-dUTP (Roche) for 1 hour at 37°C. After incubation, sections were washed with PBS and stained with nuclear dye 4′,6-diamidino-2-phenylindole (DAPI, Sigma) for observation.

### Cell Culture and pLenti6.3-PRDX6-IRES-EGFP Transfection

Moorfields/Institute of Ophthalmology-Müller 1 (MIO-M1) cells were donated by Dr. Limb (Moorfields Eye Hospital, UK). Cells were grown at 37°C in 5% CO2 in DMEM (MediaTech CellGro, Herndon, VA) with 10% fetal bovine serum, adding 2 mmol L- glutamine and 50 μg/ml gentamicin. PRDX6 was cloned out of human cDNA by PCR (primers: PRDX6-PacI-F CTGTCATTAATTAAGCCACCATGCCCGGAGGTCTGCTTC and PRDX6-AscI-R TCTACGGCGCGCCTTAAGGCTGGGGTGTGTAGCG). The PCR product and vector pLenti6.3- IRES-EGFP were digested with Pac1/Asc1 and ligated at room temperature for 4 hours. Ligation and orientation were verified by sequence analysis. Empty vector or vector containing PRDX6, which could show green fluorescence were transfected into MIO-M1 cells. Western blot and real time PCR proved PRDX6 overexpression at protein and mRNA level.

### Treatment of H_2_O_2_/Glucose Oxidase and the Survival Ratio Evaluation

Three MIO-M1 cell groups (including Lenti-PRDX6-GFP cells, Lenti-GFP cells and blank cells) were treated with H_2_O_2_/glucose oxidase (GO) at varied concentrations and exposure times. Survival ratio of each group at each condition was tested by MTS (3-(4,5-dimethylthiazol-2-yl)-5-(3-carboxymethoxyphenyl)-2-(4-sulfophenyl)-2H-tetrazolium) kit (Promega, USA). MTS (2 mg/mL; pH 6.5) was dissolved in PBS and filter sterilized. A 3 mM PMS (phenazine methosulfate) solution was also prepared (in PBS) and filter sterilized. These solutions were stored at −20°C in light-protected containers. To enhance the cellular reduction of MTS, PMS was added to MTS immediately before use (MTS-PMS ratio: 1∶20). A portion of the mixture (150 μL) was added to each well. After incubation at 37°C in a humidified atmosphere with 5% CO_2_ for 2 hours, 100 μL of the supernatant was diluted in 1 mL deionized water. The optical density was measured at 490 nm by means of spectrophotometry. Survival ratio of MIO-M1 was analyzed by means of MTS assay.

### Statistical Analysis

SPSS 18.0 (IBM Corporation, Armonk, NY) was used for statistical analysis and Origin 8.0 (OriginLab Corporation, Northampton, MA) for drawing figures and tables. Paired sample t-test and one-way ANOVA with Bonferroni correction was used for comparison between two groups and among three groups for measurement data. Differences were defined as significant at p<0.05.

## Results

### Electroretinography

**Figure 1:**
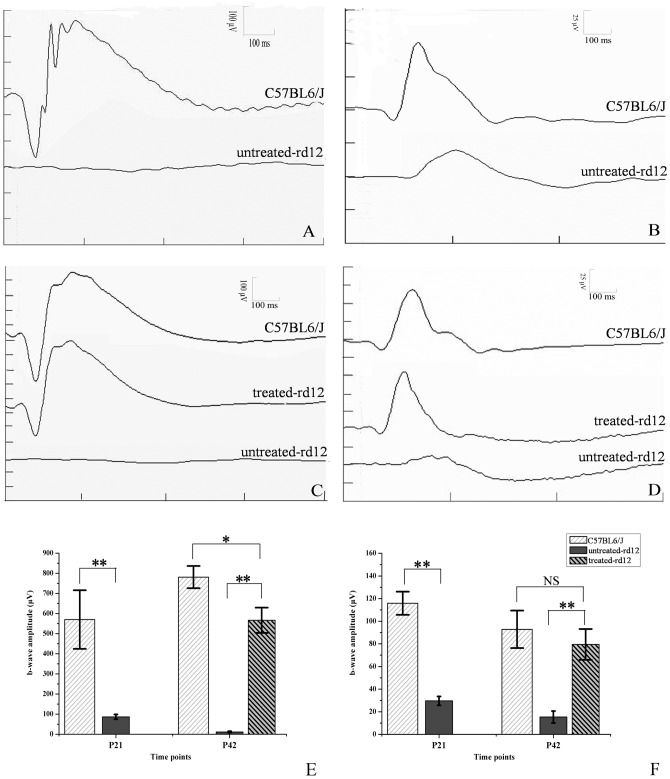
Scotopic and photopic ERG testing of untreated, treated *rd12* and normal C57BL/6J eyes. Panels A and B represent scotopic (panel A) and photopic (panel B) ERGs recorded on P21 from C57BL/6 (upper trace) or untreated *rd12* mice (lower trace). Panels C and D represent scotopic ERG (panel C) and photopic ERG (panel D) recorded on P42 from C57BL/6 (upper trace), treated *rd12* mice (middle trace) or untreated *rd12* mice (lower trace). Panels E and F indicate statistical comparison between b-wave amplitudes of different groups at both time points under scotopic and photopic conditions, respectively. N = 6/group, and error bars depict the standard error of the mean. Scotopic ERGs (A and C) were obtained using white stimulus of −5 cd·s/m^2^, while photopic ERGs (B and D) were recorded using white stimulus of 1.96 cd·s/m^2^ of *rd12* and C57BL/6J eyes. Scotopic ERG, b-wave amplitude of the untreated rd12 eye was 483.7±59.9 µV (p<0.001), lower than that of the normal control C57BL/6J eyes at P21, b-wave amplitude of the treated *rd12* eye became 555.1±28.4 µV (p<0.001) on P42, higher than that of the untreated *rd12* eye. In photopic ERG, b-wave amplitude gap was 86.2±4.54 µV (p<0.001) between the untreated *rd12* and B6 eyes on P21, and was 64.2±6.5 µV (p<0.001) between the treated and untreated *rd12* eyes on P42. NS: no significance. *: p<0.05; **: p<0.001.

In both scotopic and photopic ERGs, b-wave amplitudes of the untreated *rd12* eyes were lower than those recorded from the eyes of normal control C57BL/6J mice, with delayed b-wave peak times (Fig. 1A, 1B). ERG b-wave amplitude and peak times improved significantly in *rd12* eyes 4 weeks after P14 treatment, approaching values recorded from normal, age-matched C57BL/6J eyes (Fig. 1C, 1D). Scotopic ERG showed that a-wave amplitudes of the untreated *rd12* eyes were 9.13±3.94 µV (4.3% of normal) and 5.51±4.23 µV (1.9% of normal) on P21 and P42, respectively, whereas the treated eyes demonstrated amplitudes of 197.52±45.65 µV on P42 (68% of normal), closer to the amplitudes recorded from C57BL/6J eyes at the same age (289.50±25.15 µV). Similarly, b-wave amplitudes recorded from the untreated *rd12* eyes were 86.52±11.95 µV (15.2% of normal) and 11.60±3.38 µV (1.5% of normal) on P21 and P42, respectively, much lower than values of 570.17±146.06 µV and 781.00±55.41 µV recorded from the C57BL/6J eyes; meanwhile b-wave amplitudes recorded form the treated *rd12* eyes were 566.67±63.08 µV on P42 (72.5% of normal) (Fig. 1E). Photopic ERG signals showed similar trend (Fig. 1F). Photopic b-wave amplitude recorded from eyes of C57BL/6J mice was 86.2±4.54 µV (p<0.001, on P21) and 77.5±6.7 µV (p<0.001 on P42) greater than that recorded from untreated *rd12* eyes, while photopic b-wave amplitude recorded from treated *rd12* eyes recovered and was not significantly different from the amplitude recorded from C57BL/6J eyes on P42 (difference of 13.3±9.58 µV, p = 0.196). Thus, eyes from treated *rd12* mice displayed dramatic improvement in both scotopic a- and b-wave amplitudes and photopic b-wave amplitudes after treatment, proving that gene therapy restored retinal function.

### Retinal protein profiling by two-dimensional gel electrophoresis

**Figure 2:**
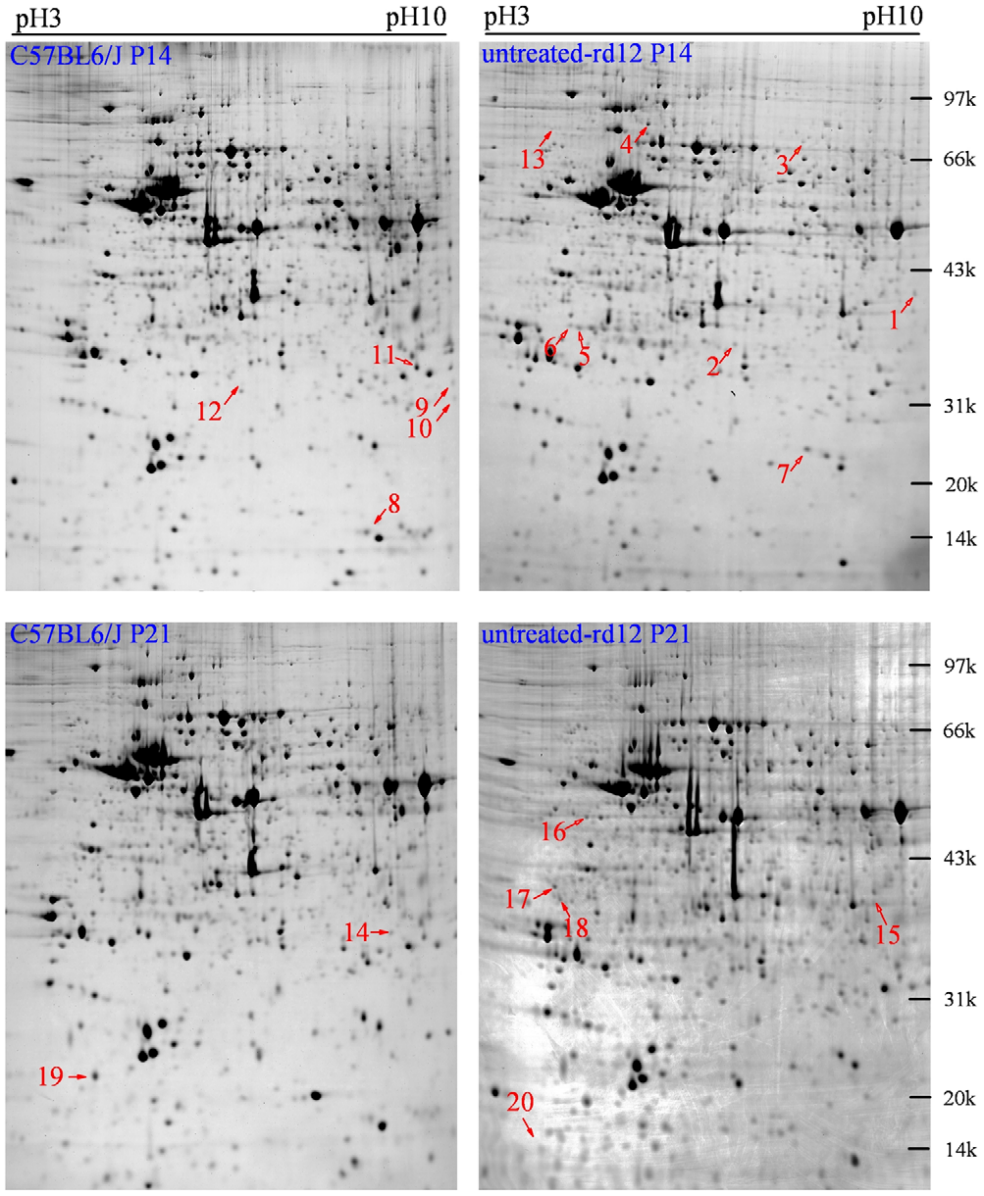
Differential retinal protein markings at age P14 and P21. Upper panels represent 2-DE gels from age P14 for C57BL/6J and untreated *rd12* mice. Lower panels represent 2-DE gels from age P21 for C57BL/6J and untreated *rd12* mice. Data are a composite of 3 independent experiments, and n = 6/group. Red numbers indicate differential proteins. At age P14, 13 differential proteins (numbered from 1 to 13) were identified, including 8 up-regulated and 5 down-regulated proteins; at age P21, 7 differential proteins (numbered from 14 to 20) were identified, including 5 up-regulated and 2 down-regulated proteins.

**Figure 3:**
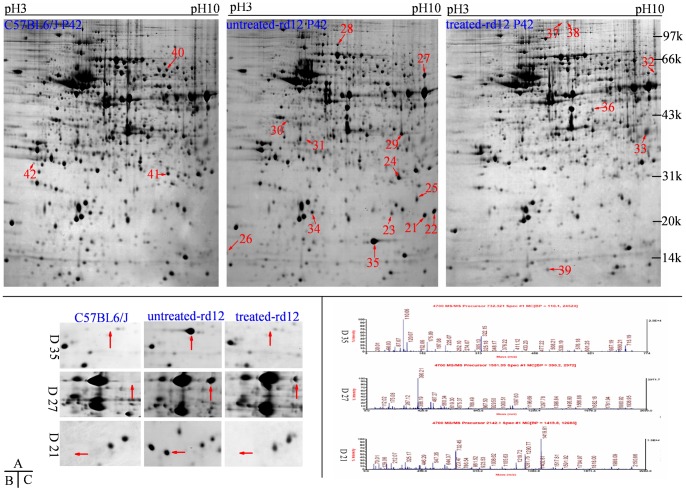
Differential retinal proteins at age P42. Upper panels (A) represent 2-DE gels of wide-type C57BL/6J (left panel), untreated *rd12* (middle panel) and treated *rd12* eyes (right panel) at age P42. Data are a composite of 3 independent experiments, and n = 6/group. Differential proteins are marked with red numbers (from 21 to 42). Magnified details of differential proteins No. 21, 27 and 35 among the 3 eye groups at age P42 are presented in **B**. Graphical representation of mass spectrometry peaks from differential proteins No. 21, 27 and 35 are shown in **C** (the x- and y-axis represent mass-to-charge ratio (m/z) and relative intensity, respectively; the mass numbers of monoisotopic peaks [M+H]+ for peptides are marked above individual peaks).

Two-dimensional gel electrophoresis proteomic maps of retinal protein samples of the 3 groups were generated using 17 cm gels over a pH range of 3.0–10.0. Image analysis by Image Master 2D determined the presence of ∼1600 protein spots on every gel, most of which were distributed in a pH range of 4.0–8.0. Three repeats were performed on every sample and the analysis of the repeats demonstrated high repeatability. Comparisons on P14, P21 were made between untreated *rd12* and normal control retinas, and those on P42 were among treated and untreated *rd12* retinas and control retinas. On P14, 13 differential spots were identified including 8 up-regulated and 5 down-regulated proteins; on P21, 7 spots were considered differential, with 5 up-regulated and 2 down-regulated (Fig. 2). On P42, apparent difference in protein expression displayed between the untreated and the treated *rd12* eyes; 22 differentially expressed protein spots were identified and further investigated by the mass-spectrometry (MALDI-TOF-MS/MS) analysis searching against NCBInr database (Fig. 3C). Among these, 13 proteins had over expression in the untreated *rd12* group versus the treated *rd12* and C57BL/6J groups; meanwhile, the treated *rd12* group had 6 up-regulated and 3 down-regulated proteins versus the C57BL/6J group. In summary, a total of 42 differential proteins with high repeatability were selected for MALDI-TOF-MS/MS analysis.

### Mass spectrometry analysis

Of the 42 proteins tested, 41 demonstrated mass spectrum peaks (except protein 12). Successful matches were achieved in 39 cases by mascot software with a matching ratio of 99%–100%. The information from MALDI-TOF-MS/MS analysis, including the name, NCBI number, theoretical isoelectric point and molecular weight, number of matched peptide and coverage, is listed in Table 2 and Table 3. Table 2 lists the 19 identified differential proteins between *rd12* mice and C57BL/6J mice retinas at age P14 and P21 (protein 12 was not identified), while Table 3 shows the other 22 differential proteins at age P42.

**Table 2:** Mass spectrometry results of retinal differential proteins between rd12 and C57BL/6J mice at age P14 and P21.

Group ID	Protein	Acc.no.	Theoretical		No.of matched peptides	Coverage (%)	Protein Score
			MW (kDa)	PI			
1	adipo Q ↑	gi|1399498	26.83	5.3	5	24.41	37
2	UNC-119 homolog ↑	gi|6755939	26.99	5.81	24	81.24	767
3	dihydropyrimidinase-like 3 isoform 2 ↑	gi|6681219	61.9	6.04	22	50.96	395
4	dynein cytoplasmic 1 intermediate chain 2 ↑	gi|6753658	68.35	5.16	13	58.81	242
5	PR264/SC35 ↑	gi|1405747	13.97	10.24	12	66.38	275
6	unnamed protein product ↑	gi|26327249	29.7	5.15	14	27.88	218
7	similar to splicing factor,arginine/serine-rich 3 ↑	gi|224084730	14.2	10.12	13	64.24	292
8	unnamed protein product ↓	gi|12846244	17.97	8.44	7	72.52	422
9	voltage-dependent anion channel 1 ↓	gi|6755963	30.74	8.62	9	7.09	97
10	platelet-activating factor acetylhydrolase ↓	gi|6679201	25.84	6.42	13	89.24	338
11	Wbscr1 alternative spliced product ↓	gi|4972951	25.17	7.79	7	70.63	228
13	unnamed protein product ↑	gi|26329075	47.02	4.68	11	72.02	430
14	endoplasmic reticulum protein ERp29 precursor ↓	gi|19526463	28.81	5.9	8	22.37	197
15	cytosolic malate dehydrogenase ↑	gi|387129	36.45	6.16	14	73.3	608
16	keratin 15 ↑	gi|226823220	49.46	4.79	28	80.67	669
17	unnamed protein product ↑	gi|12833697	32.8	4.64	21	47.21	355
18	alpha-tropomyosin ↑	gi|157787199	32.66	4.69	29	42.98	425
19	ribosomal protein, large P2 ↓	gi|83745120	11.64	4.42	8	59.48	540
20	phosphoprotein enriched in astrocytes 15 isoform 2 ↑	gi|21426847	15.04	4.94	7	51.22	315

Note: ↑: the protein level is up-regulated in the untreated *rd12* retinas; ↓: the protein level is down-regulated in the untreated *rd12* retinas.

**Table 3:** Mass spectrometry results of retinal differential proteins between wide-type C57BL/6J, untreated rd12 and treated rd12 eyes at age P42.

Group ID	Protein	Acc.no.	Theoretical		No.of matched peptides	Coverage (%)	Protein Score
			MW (kDa)	PI			
21	peroxiredoxin 6 ↑	gi|6671549	24.81	5.98	16	89.78	777
22	crystallin, alpha A ↑	gi|30794510	22.48	6.35	15	72.68	395
23	crystallin, beta A4 ↑	gi|10946672	22.45	5.9	13	78.39	586
24	crystallin, beta A1 ↑	gi|20304089	25.19	5.98	19	86.31	593
25	crystallin, beta A1 ↑	gi|20304089	25.19	5.98	15	79.92	570
26	ribosomal protein, large P2 ↑	gi|83745120	11.64	4.42	9	58.13	484
27	Heat shock protein 70 ↑	gi|42542422	70.83	5.28	33	79.74	674
28	hypothetical protein LOC433182 ↑	gi|70794816	47.11	6.37	29	59.92	726
29	crystallin, beta A1 ↑	gi|20304089	25.19	5.98	16	85.66	475
30	eukaryotic translation elongation factor 1 delta isoform b ↑	gi|54287684	31.27	4.96	11	30.94	212
31	unnamed protein product ↑	gi|26328639	17.82	9.41	11	61.51	409
32	fascin homolog 1, actin bundling protein *	gi|113680348	54.47	6.44	26	35.72	321
33	peroxiredoxin 6 *	gi|6671549	24.81	5.98	24	89.54	837
34	modifier 2 ↑	gi|53165	19.74	4.96	13	72.23	571
35	alpha-A-crystallin ↑	gi|387134	18.53	5.86	16	75.91	499
36	L-lactate dehydrogenase B *	gi|6678674	36.55	5.7	13	52.44	311
37	Spna2 protein *	gi|20380003	156.1	5.29	17	17.88	52
38	Spna2 protein *	gi|20380003	156.1	5.29	45	43.65	259
39	retinol binding protein 1, cellular *	gi|6755300	15.84	5.1	14	86.17	494
40	RecName: Full = Inosine-5′- monophosphate dehydrogenase 1; Short = IMP dehydrogenase 1; AltName: Full = IM #	gi|1708472	55.26	6.29	23	56.13	541
41	crystallin, beta A2 #	gi|10946978	22.22	6.3	12	73.53	617
42	eukaryotic translation initiation factor 6 #	gi|27501448	26.49	4.63	5	22.4	264

Note: ↑: the protein is up-regulated in the untreated *rd12* retinas; *: the protein is up-regulated in the treated *rd12* retinas; #: the protein is up-regulated in the C57BL/6J retinas.

### Verification of differential proteins

Of the 39 successfully matched differential proteins, alpha A crystallin (CRYAA, a molecular chaperone), heat shock protein 70 (HSP70) and peroxiredoxin 6 (PRDX6), were considered as possible candidates implicated in the degenerative process of LCA. To clarify this hypothesis, additional tests related to these 3 proteins were performed.

### Immunofluorescence

**Figure 4:**
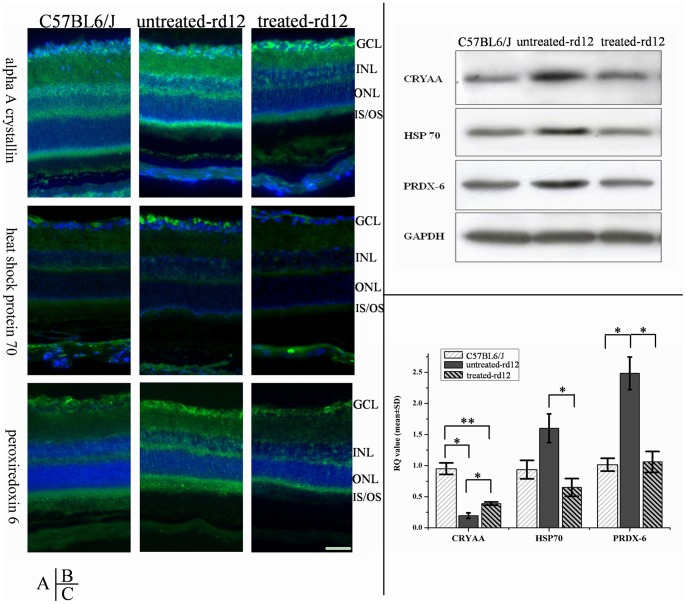
Immunofluorescence, Western blot and RT-PCR of CRYAA, HSP70 and PRDX6. Immunofluorescence (**A**), western blot (**B**) and RT-PCR (**C**) results of CRYAA, HSP70 and PRDX6 in retinas of wide-type, untreated *rd12* and treated *rd12* eyes at P42. Data are a composite of 3 independent experiments (for A, B and C), and n = 6/group (B) and 4/group (C). The green staining in **A** represents the targeted proteins (FITC) while the blue color indicates nucleus staining with DAPI. Western blot (**B**) confirmed that all 3 proteins had higher expressions in untreated *rd12* group, while the difference between the treated *rd12* and C57BL/6J group was not significant. RT-PCR results (**C**) showed that CRYAA mRNA level was down-regulated in all 3 groups, and the untreated *rd12* group demonstrated the lowest levels. HSP70 mRNA levels of the untreated *rd12* group were 2 times higher compared to the treated *rd12* group (p<0.05), and 1.6 times higher compared to the C57BL/6J group (p>0.05). PRDX6 mRNA of the untreated *rd12* group was 2.4 times higher compared to the other 2 groups (p<0.05), while the mRNA levels of the treated *rd12* group were not different compared to that of the C57BL/6J group. Error bars depict the standard error of the mean. Calibration bar  = 50 μm.

Immunofluorescence showed that CRYAA was expressed in mouse retina from all 3 groups on P42, and it was mainly distributed in the ganglion cell layer and the outer nuclear layer, with a weak expression in both inner and outer plexiform layers. Compared to the treated *rd12* and C57BL/6J groups, CRYAA of the untreated *rd12* group displayed stronger expression in ganglion and photoreceptor cells (Figure 4A, upper panels).

HSP70, which was localized predominantly in the ganglion cell layer (but also in the RPE layer), also showed much stronger expression in the untreated *rd12* group than that in C57BL/6J and treated *rd12* groups on P42 (Figure 4A, middle panels).

PRDX6 had an extensive expression in every retinal layer and was also over expressed in the untreated *rd12* mice group compared to the other two groups on P42 (Figure 4A, lower panels).

In summary, the immunofluorescence results confirmed and expanded the information about those 3 retinal proteins obtained from the 2-DE analysis.

### Western blot

Western blot was used to analyze the differences in the 3 selected proteins semi-quantitatively, and confirmed that the expression of all 3 proteins (CRYAA, HSP70 and PRDX6) in the untreated *rd12* group was much stronger than that in the treated *rd12* and C57BL/6J groups, while their expressive intensity in the latter 2 groups was quite similar (Fig. 4B).

### RT-PCR

Real-time PCR was conducted to obtain information at mRNA level, which showed that CRYAA mRNA was down-regulated in all 3 groups, the untreated *rd12* group demonstrating the lowest production (Fig. 4C). HSP70 mRNA level in the untreated *rd12* eyes was up-regulated and 2 times higher than that of the treated *rd12* group (p<0.05), and 1.6 times higher than that of the C57BL/6J group (p>0.05). PRDX6 mRNA expression in the untreated *rd12* group was 2.4 times higher than that in the other 2 groups (p<0.05), while the level in the treated *rd12* group was not different from that in the C57BL/6J group (p>0.05).

### Reactive Oxygen Species and apoptosis

**Figure 5:**
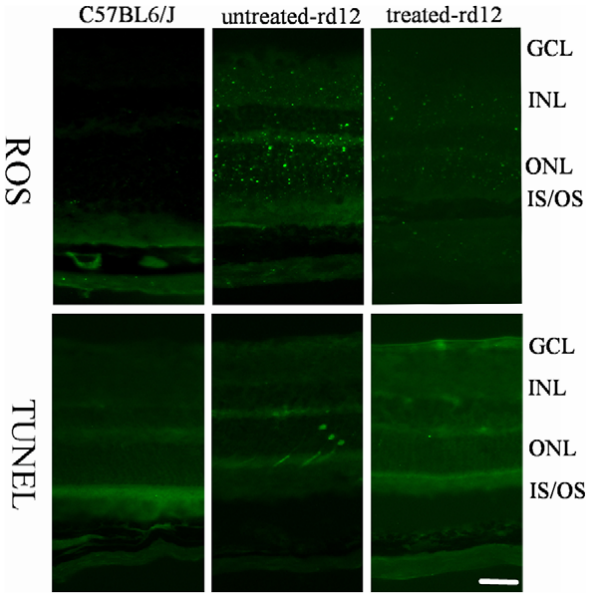
Reactive oxygen species and apoptosis determination at age P42. Green fluorescent staining indicates expression of ROS in the upper row panels, while it indicates apoptotic cell death by TUNEL in the lower row panels. Data are a composite of 3 independent experiments. Note the dramatic decrease in ROS presence in treated *rd12* eyes (right upper panel) compared to the abundant presence of ROS in the untreated *rd12* eyes (middle upper panel). Similarly, no signs of apoptosis are present in the treated *rd12* eyes (lower right panel), and several apoptotic cells could be identified in the untreated *rd12* eyes (middle lower panel). Calibration bar  = 50 μm.

The distribution and expression pattern of retinal ROS was detected by ROS fluorescein kit and confocal microscopy. ROS expression was stronger in untreated *rd12* eyes compared with C57BL/6J eyes, while the expression of ROS in treated *rd12* eyes decreased dramatically, approaching normal levels (Fig. 5, upper panels). A TUNEL test was used to evaluate retinal cell apoptosis. No signs of apoptosis were detected in C57BL/6J and treated *rd12* groups on P42, while in the untreated *rd12* group, several apoptotic cells could be localized at higher magnification. (Fig. 5, lower panels).

### Cell Culture and Lenti-PRDX6-GFP Transfection

Recombinant lentiviral vector with PRDX6 was constructed to infect Müller cells in culture in order to investigate the effect of PRDX6 overexpression, and its role in the process of oxidative damage. By transfecting Lenti-GFP into MIO-M1 cells, the best transfective condition was tested to be MOI  = 5 with polybrene addition. After transfection of Lenti-PRDX6-GFP, PCR confirmed that PRDX6 gene was recombinant to the host gene, and gene sequencing verified that it was 100% identical to the human PRDX6.

**Figure 6:**
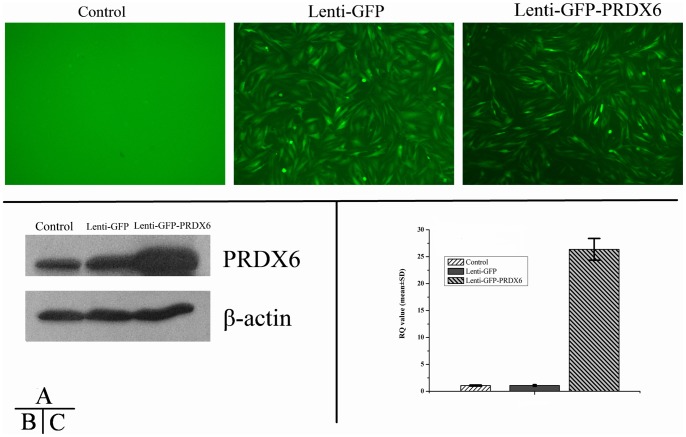
Immunofluorescence and Western blot of Lenti- PRDX6-GFP vector in MIO-M1 cells. Transfection of Lenti-PRDX6-GFP was evaluated by immunofluorescence (**A**), western blot (**B**) and RT-PCR (**C**). Data are a composite of 6 independent experiments for A, and 3 for B and C. Panels in **A** represent confocal micrographs and demonstrate GFP immunofluorescence in transfected groups indicating successful transfection; Western blot results in **B** indicate that PRDX6 group expressed much more PRDX6 than either the GFP or the blank group at protein level; Bar graphs in **C** indicate the results from the RT-PCR analysis, demonstrating an over expression of PRDX6 6.3 times compared to the other two groups at mRNA level (p<0.01). Error bars depict the standard error of the mean. Calibration bar  = 50 μm.

Seventy-two hours after transfection of Lenti-GFP and Lenti-PRDX6-GFP, MIO-M1 cells demonstrated intensive green fluorescence inside the cell bodies, which indicated successful transfection (Fig. 6A, central and right panel). Western blot confirmed that Lenti-PRDX6-GFP group expressed much more PRDX6 than the Lenti-GFP or blank (control) group at a protein level (Fig. 6B); RT-PCR demonstrated that the mRNA levels in the PRDX6 group were 6.3 times higher than that in GFP group or the blank (control) group (p<0.01; Fig. 6C). There was no statistically significant difference on mRNA levels between the GFP and the blank (control) group, confirming that vector transfection did not affect PRDX6 expression in normal cells.

### Survival Ratio Evaluation after Treatment with H_2_O_2_ or Glucose Oxidase

**Figure 7:**
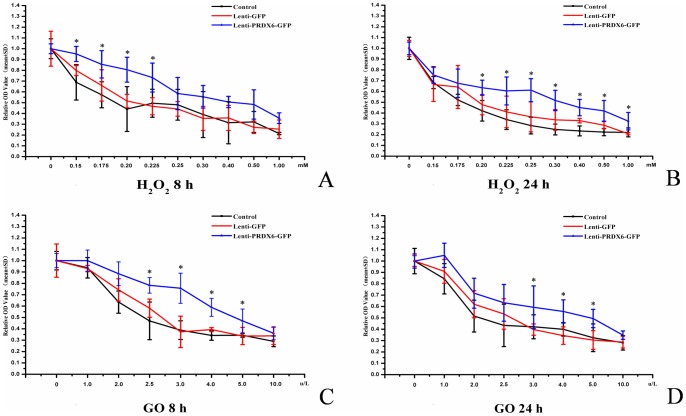
Survival rate of MIO-M1 cells after administration of H_2_O_2_ and GO and at varied concentrations and time points. Panels **A** and **B** represent the relative optical density (OD) values (positively related to number of live cells) of surviving MIO-M1 cells at 8 hrs (**A**) and 24 hrs (**B**) at various concentrations of H_2_O_2_ (in mM – horizontal axis). Panels C and D represent average values (±SEM) of surviving MIO-M1 cells at 8 hrs (**C**) and 24 hrs (**D**) at various concentrations of GO (in µg/L – horizontal axis). N = 6/group in every panel, error bars depict the standard error of the mean. Results showed that the survival ratio of the PRDX6 group was always higher than those in the GFP and control group after treatment of H_2_O_2_ and GO at varied concentrations for 8 and 24 hours. The concentration range of H_2_O_2_ treatment, where significant difference was observed between the PRDX6 group and the GFP or the blank group, was from 0.15 to 0.225 mM at 8 hours, and from 0.20 to 1.00 mM at 24 hours (p<0.05). After GO treatment, the concentration range where significant difference was observed ranged from 2.5 to 5.0 µg/L at 8 hours, and from 3.0 to 5.0 µg/L at 24 hours (p<0.05).

Survival of MIO-M1 cell decreased in a concentration-dependent way after treatment with H_2_O_2_. However, cells transfected with Lenti-PRDX6-GFP consistently demonstrated higher survival rate compared to either the Lenti-GFP group or the blank (control) group (Fig. 7A, 7B). For example, 8 hours after 0.225 mM H_2_O_2_ treatment, the survival ratio of MIO-M1 cells decreased to 50% in Lenti-GFP and blank (control) groups, compared to 80% in the Lenti-PRDX6-GFP group. For all groups, the survival rate continued to decrease with prolonged exposure and increased concentration of H_2_O_2_. Of note, the survival ratio of the Lenti-PRDX6-GFP group was always higher than that of the other two groups, and the difference was statistically significant in the range from 0.150 to 0.225 mM at 8 hours, and from 0.200 to 1 mM at 24 hours (p<0.05) (Fig. 7A, 7B).

The effect of GO application on MIO-MI cell survival was similar to that of H_2_O_2_ in the three groups. The survival rate of the Lenti-PRDX6-GFP group treated with GO was always higher than that in the other two groups (Fig. 7C, 7D); however, statistically significant difference was observed only in the range from 2.5 to 5.0 µg/L at 8 hours, and from 3.0 to 5.0 µg/L at 24 hours (p<0.05).

## Discussion

### Gene therapy in the rd12 mouse

Our results demonstrated that scotopic ERG responses were abnormally low at age P21 and became undetectable at age P42 in the untreated *rd12* mice, similar to our previous reports [32]. After subretinal administration of scAAV5-smCBA-hRPE65 vector, ERG amplitudes recovered dramatically to a level close to that of age-matched wild-type mouse, indicating a positive effect of gene therapy on retinal function, consistent with our previous observation at a slightly older age [30]. Scotopic ERG or photopic ERG was recorded in our previous reports [30], [32], while in this present study both types of ERG on the same mice were recorded and dramatic recovery was found for both rods and cones. The observation that gene therapy is protective to the cone function is an important extension of our previous findings, as it indicates the potential for restoration of photopic vision in LCA patients, which is much more related to their daily activities and quality of life.

Previous study by our group demonstrated that RPE65 protein expression was positively correlated with improved ERG activity after the administration of the same vector scAAV5-smCBA-hRPE65 in *rd12* mice [30]. Although rod outer segments degeneration goes on, outer nuclear layer appears normal at P42 in *rd12* eyes; meanwhile, cone degeneration starts around P14 and only a small number of M-cones remain at the dorsal and temporal parts of the *rd12* retina at age P42 [32], [33]. These findings focused on investigation of morphological changes in the retinal structure before and at age P42, however much more vital information might be explored by proteomic changes in this period, when the retinal function had already significantly deteriorated while the morphology still preserved relatively. The present study complemented this deficiency by conducting a retinal proteomic analysis in *rd12* mice at ages P14, P21 and P42, after a subretinal injection of the scAAV5-smCBA-hRPE65 vector at P14.

### Proteomic differences

Currently, very little is known about the proteomic makeup in animal models of inherited retinal degenerative disorders (RDD). Cavusoglu et al. (2003) found loss of proteins involved in the rod-specific phototransduction cascade and induction of proteins from the crystallin family in rd1 mice, with a recessive mutation in the *PDE6B* gene [34]. Finnegan et al. (2010) observed several proteins differentially expressed during retinal degeneration in chicken model of retinal degeneration [35]. LCA is a multigenic retinal degenerative disorder which may involve various protein alterations during the disease progression and currently there is a lack of published data about retinal protein changes during the natural course of the disease or related to any treatment paradigms in either animal models or in the human condition.

Apart from three unsuccessfully identified proteins, our results identified a total of 39 differential proteins between the treated, untreated and C57BL/6J retinas at age P14, P21 and P42. Surprisingly, the proteins that were differential at age P14 or P21, were not differential at P42. This result suggests a protein expression and regulation pattern that changes considerably during the age range investigated in this study.

Of note, the crystalline family was well represented among the differential proteins at age P42. Seven (32%) out of the 22 differential proteins identified at this time point were members of this family (Table 3). Although crystallins are usually considered structural proteins found in the lens and the cornea of the eye, recent researches showed that they were involved in RDD [36], [37]. The alpha crystallin forms are similar to the small heat shock proteins with chaperone-like properties, including the ability to increase cellular tolerance to stress. Therefore, we decided to further investigate the role of alpha A crystallin (CRYAA) in *rd12* mice. Similarly, heat shock protein 70 (HSP70) was of a particular interest in the set of identified differential proteins, as it has been demonstrated that its expression declines during normal aging of the retina [38] and is involved in retinal resistance to injury either in vitro [39] or in vivo, in a mouse model of LCA [40]. Finally, in this set of proteins, there is a lack of information about the role of peroxiredoxin-6 (PRDX6) in RDD. However, it has been demonstrated that PRDX6 is involved in glutamate-induced retinal toxicity [41], [42] and protects against hypoxia-induced retinal ganglion cell damage [43]. Thus, the detailed investigation of PRDX6 was considered important and this protein was also selected for additional tests.

### Alpha A crystallin

The alpha A crystalline protein, was originally found in lens tissue and its role was established as one of the main factors maintaining the transparency of the vertebrate lens [44]. Subsequently, its presence was identified in other cells types and ocular tissues. In 1994, Deretic et al. reported that alpha A- and alpha B-Crystallins bound specifically to the photoreceptor post-Golgi membranes that mediated transport of newly synthesized rhodopsin in frog retinal photoreceptors [45]. This protein was also demonstrated to be a protective factor in retinal pathologic processes, as mRNA of CRYAA as well as other 9 crystallins displayed strong sustained up-regulation after retinal [46] and light injury [47]. In the present study, we found that CRYAA levels were significantly higher in the untreated *rd12* group compared to the treated *rd12* and control (C57BL/6J) groups. The precise mechanism of CRYAA involvement in the process of retinal degeneration is currently unknown. Our previous findings showed that rod outer segments in *rd12* mice are degenerated and many cones are dead at P42 [32]. One possibility is that the elevated CRYAA in untreated *rd12* retinas was a response to photoreceptor cell death, helping the RPE cells to phagocyte the degenerating photoreceptor outer segments.

There was a discordance in CRYAA expression between the mRNA and protein level in untreated *rd12* at age P42 (compare CRYAA levels in Fig. 4B and 4C). This might be caused by a negative feedback between protein levels and mRNA expression, as the elevated levels of retinal CRYAA protein might inhibit mRNA transcription to maintain level of the protein in the tissue within certain range.

### Heat shock protein 70

Heat shock protein 70 is a member of the heat shock protein family, which is an important part of the process of proper protein folding, and helps protect cells from stress [48]. It also functions as an intracellular chaperone for other proteins, assisting in the establishment of proper protein shape, disposal of damaged or defective proteins and facilitating the transmembrane transport of proteins [49]. By influencing multiple steps in apoptotic signaling cascades, HSP70 is a powerful anti-apoptotic protein [50], and was also found to be neuroprotective in the context of retinal ganglion cell injury by an interaction with HSP27 [51].

In our experiments, HSP70 was localized mainly in the ganglion cell layer and less in the RPE layer. The protein mRNA level in untreated *rd12* eyes was much higher than that in either treated *rd12* eyes or normal eyes. The normalization of HSP70 level in treated *rd12* eyes (both at a protein and mRNA levels) correlates well with our observation that retinal apoptosis is not present in those eyes in contrast to the untreated *rd12* eyes. This finding indirectly supports previous studies demonstrating that HSP70 could play an inhibitory role in retinal degeneration by protecting the neuronal cells from stress-induced apoptosis [51].

### Peroxiredoxin, reactive oxygen species and apoptosis

Peroxiredoxins are a ubiquitous family of antioxidant enzymes that also control cytokine-induced peroxide levels and thereby mediate signal transduction in mammalian cells [52]. Peroxiredoxins share the same basic catalytic mechanism, in which a redox-active cysteine (the peroxidatic cysteine) in the active site is oxidized to a sulfenic acid by the peroxide substrate [53], and they have recently been of elevated scientific interest due to their distinctive role in the disposal of ROS and peroxide. Peroxiredoxin-6 (PRDX6), one particular member, has been found to provide a neuroprotective effect from glutamate, TNF-alpha and hypoxia induced cytotoxicity by reducing ROS level and NF-κB activation in rat retinal ganglion cells (42, 43). Kubo et al. found abundant protein supply of PRDX6 and PRDX5 inhibited the oxidative stress-induced DNA damage after high-glucose exposure in retinal pericytes [54].

Similar to the results of HSP70, the expression of PRDX6 in untreated *rd12* retinas was also found to be much higher than that in normal age-matched or the treated retinas, which supported the previous findings of PRDX6 as a neuroprotector from retinal oxidative stress. PRDX6 level in treated *rd12* retinas was also close to that of normal C57BL/6J retinas, which was validated both at protein and mRNA levels. The normalization of PRDX6 level in treated *rd12* retinas is an additional demonstration of the effectiveness of gene therapy. In addition, tests of ROS expression confirmed their presence in retinal photoreceptors and elevated levels in untreated *rd12* eyes, indicating that over expression of PRDX6 protected retina from oxidative damage by removal of ROS. TUNEL tests confirmed no obvious signs of retinal cell apoptosis in treated *rd12* mice and in C57BL/6J mice. In contrast, a low level of apoptosis was observed in untreated *rd12* eyes at age P42.

Oxidative damage has been found to play an important role in vision-threatening retinal diseases such as age-related macular degeneration. There are multiple types of ROS scavengers in the retina, including PRDXs [55], to resist high level of ROS which induce neuronal dysfunction [56]. ROS accumulation was observed in models of retinal light damage, in which retinoid visual cycle was affected [57]. In *rd12* retina, the absence of *RPE65* suppresses the retinoid cycle and probably induces an increase in ROS. After application of *RPE65* gene therapy, the increased *RPE65* expression likely restored the retinoid visual cycle and enabled regenerated 11-*cis*-retinal to conjugate with its apoprotein, opsin, thus eliminating the main cause of photoreceptor cell death in *rd12* retinas. Most likely, this is the reason why ROS, as well as other protective factors (CRYAA, HSP70 and PRDX6), decreased to a normal level in treated *rd12* eyes.

With gene recombinant technique, the increase of PRDX6 expression resulted in a considerable reduction of H_2_O_2_-induced oxidative damage. When the H_2_O_2_ concentration exceeded certain threshold, this resulted in very high levels of cell death, indicating that antioxidative effect of PRDX6 is limited to a range of concentration of peroxides. Our findings support the evidence for an antioxidative effect of PRDX6 in the retina and suggest that normalization of PRDX6 level could be a good indicator for a decreased retinal injury due to ROS. They also support the possibility that PRDX6 could be used as an indicator of therapeutic effectiveness in models of RDD.

A weakness of the current study is the absence of data from sham injected eyes that would control for both potential growth factor release caused by wounding of the eye, as well as the effect of topical dexamethasone treatment after injection. However, the results on protein change studies revealed no identified growth factors in the injected eyes, and elevated levels of proteins which appeared to return to control levels at the end of the study period, indicating that both the wounding of the eye by the injection itself and the use of dexamethasone had minimal effect and are unlikely to be confounding factors in this case. Another limitation in the present study is that only three of the identified differential proteins have been tested and explored in more detail. The remaining proteins, especially the unnamed ones, deserve further exploration of their identities and functions. Also the mechanistic detail about the PRDX6 function in the retina and its role in the disease process is still unclear and needs more investigation.

### Conclusions

Our results support the effectiveness of gene therapy in LCA and reveal new details of disease related protein level changes. Three proteins involved in regulation of apoptosis and neuroptotection were discovered, which pointed to specific potential mechanisms of restoring retinal homeostasis.
